# Risk of acute myocardial infarction in upper tract urothelial carcinoma patients receiving radical nephroureterectomy: a population-based cohort study

**DOI:** 10.18632/oncotarget.18495

**Published:** 2017-06-15

**Authors:** Shih-Yi Lin, Cheng-Li Lin, Chao-Hsiang Chang, His-Chin Wu, I-Kuan Wang, Che-Yi Chou, Ji-An Liang

**Affiliations:** ^1^ Graduate Institute of Clinical Medical Science, College of Medicine, China Medical University, Taichung, Taiwan; ^2^ Department of Internal Medicine, China Medical University Hospital, Taichung, Taiwan; ^3^ Division of Nephrology and Kidney Institute, China Medical University Hospital, Taichung, Taiwan; ^4^ Managment Office for Health Data, China Medical University Hospital, Taichung, Taiwan; ^5^ College of Medicine, China Medical University, Taichung, Taiwan; ^6^ Department of Urology, China Medical University Hospital, Taichung, Taiwan; ^7^ Department of Radiation Oncology, China Medical University Hospital, Taichung, Taiwan

**Keywords:** upper tract urothelial carcinoma, acute myocardial infarction, mortality, radical nephroureterectomy, multivariable Cox proportional hazard regression analysis

## Abstract

**Background:**

The outcomes of upper tract urothelial carcinoma (UTUC) receiving radical nephroureterectomy were usually limited to small sample size, case-control studies, and often focused on cancer progression. Risk of acute myocardial infarction (AMI) in these patients was never investigated.

**Results:**

The overall incidences of AMI were 3.39, 1.44, and 1.70 per 10,000 person-years in the radical nephroureterectomy, nonnephroureterectomy, and non-UTUC cohorts, respectively. Multivariable Cox proportional hazard regression analysis revealed a significantly higher AMI risk in the radical nephroureterectomy cohort [adjusted HR (aHR) = 1.83, 95% confidence interval (CI) = 1.08–3.11], compared with non-UTUC cohorts. The risk of mortality were the highest in patients with UTUC who had undergone radical nephroureterectomy [adjusted HR (aHR) = 5.37, 95% confidence interval (CI) = 4.80–6.02].

**Materials and Methods:**

From the Taiwan National Health Insurance claims data, 1,359 patients with UTUC who had undergone radical nephroureterectomy and 3,154 patients with UTUC who had undergone nephron sparing surgery and were newly diagnosed between 2000 and 2010 were identified. For each patient, 4 individuals without UTUC were randomly selected and frequency matched by age, sex, and diagnosis year.

**Conclusions:**

Patients with UTUC who have undergone radical nephroureterectomy are at a higher risk of developing AMI, compared with those receiving nephron sparing surgery.

## INTRODUCTION

Despite the improvements of intervention therapy and pharmacological managements, acute myocardial infarction (AMI) remains major evens with serious consequences in increasing medical expenditure, morbidity, and mortality [1, 2]. One key to success in decreasing AMI associated complications is timely delivery of reperfusion therapy and fibrinolytic therapy [3]. Another most cost-effectiveness key is primary prevention which targets high-risk candidates. [4] Numerous risk factors of acute myocardial infarction has been identified including age, diabetes, hypertension, hyperlipidemia, metabolic syndrome, and smoking, et al. [5] However, these risk factors are usually applicable to general population. Chronic kidney disease (CKD), the results of reduced functional nephron mass, has also been recognized as a risk factor of ischemia heart disease [6, 7].

Upper tract urothelial carcinomas (UTUCs), neoplasms arising in the kidneys and ureters, accounts for 5% of all urothelial tumors [8–10]. Radial nephroureterectomy with bladder cuff excision is the gold standard treatment for patients with UTUC [11]. Reduced nephron mass, the results after nephroureterectomy, would be associated with higher rates of CKD development and end stage renal disease [10, 12, 13]. Recent years, several studies have investigated the outcomes of UTUC patients receiving radial nephroureterectomy versus nephron sparing surgery in order to decrease the ostoperative complications [14, 15]. However, whether radical nephroureterectomy versus nephron sparing surgery would cause different outcomes of AMI and total mortality are unknown. No cohort study has yet evaluated the association between radical nephroureterectomy and the incident cardiovascular events.

The National Health Insurance (NHI) database of Taiwan is a nationwide database with cohort data on 23 million people. Using the NHI data, this study determined whether patients with UTUC who have undergone radical nephroureterectomy are at high risks of AMI.

## RESULTS

**Figure 1:**
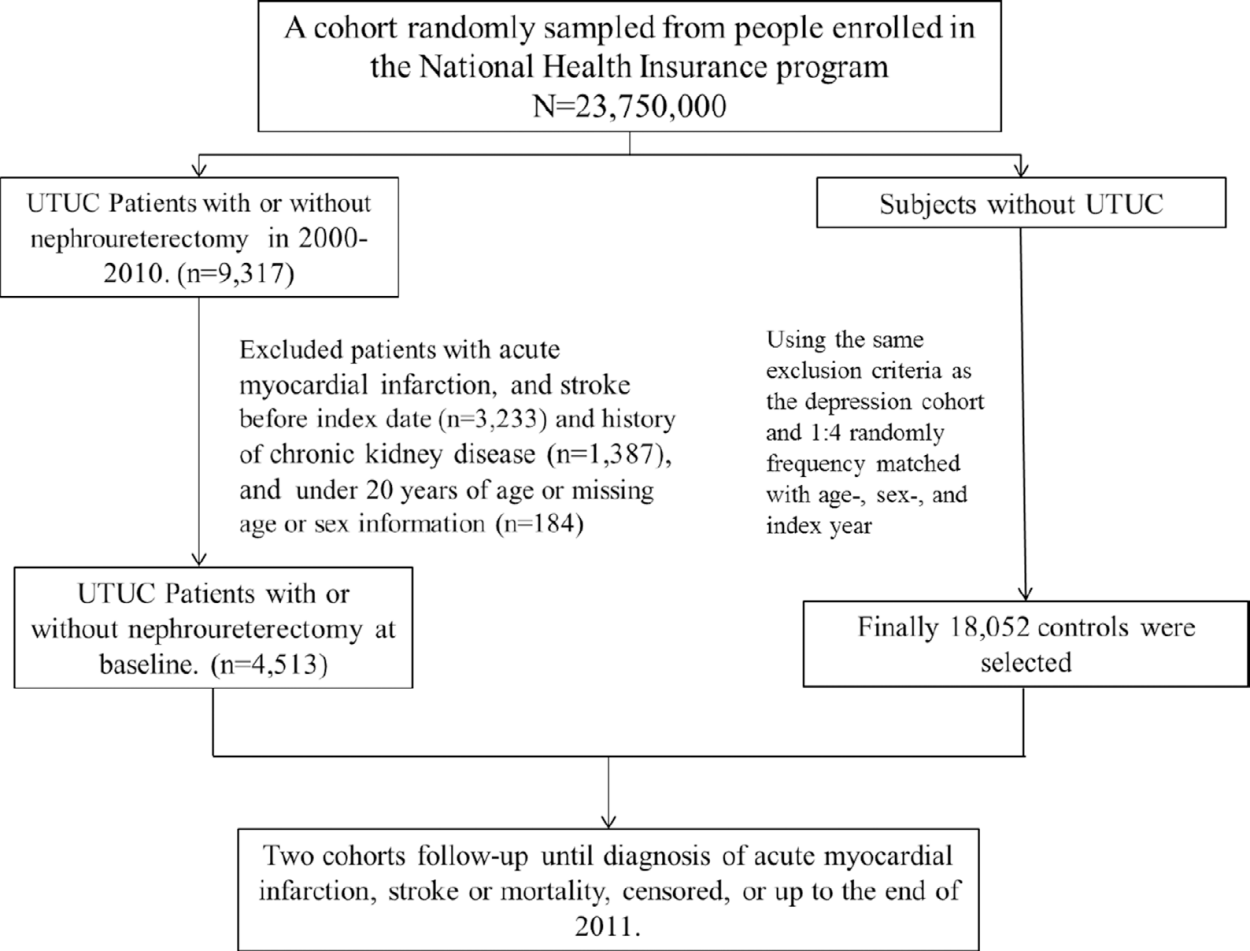
Study design and enrollment process

We identified 4,513 patients with UTUC (3,154 patients who had undergone nephron sparing surgery and 1,359 patients who had undergone radical nephroureterectomy) (Figure 1). The non-UTUC cohort comprised 18,052 subjects with similar distributions in age and sex. The mean age of the patients in the radical nephroureterectomy, nonnephroureterectomy, and non-UTUC cohorts was 66.6(± 11.8), 64.4 (± 13.3), and 65.0 (± 12.9) years, respectively. The majority of the patients (55.4%) fell into the age group of > 64 years, and there were more males (53.7%) in the study cohorts. Compared with the non-UTUC cohort, significantly higher prevalence of diabetes, hypertension, hyperlipidemia, COPD, and CAD was found in the UTUC cohorts (all *P* < 0.001) (Table 1).

**Table 1:** Comparison of demographics and comorbidity between patients with urothelial cancer and controls

			Upper tract urothelial cancer						*p*-value
	Control (*N* = 18052)		Without radical nephroureterectomy (*N* = 3154)		With radical nephroureterectomy (*N* = 1359)		Total (*N* = 4513)		
	*n*	%	*n*	%	*n*	%	*n*	%	
**Age, year**									0.99
20–49	2416	13.4	470	14.9	134	9.86	604	13.4	
50–64	5620	31.1	1008	32.0	397	29.2	1405	31.1	
> 64	10016	55.5	1676	53.1	828	60.9	2504	55.5	
Mean (SD) ^#^	65.0	12.9	64.4	13.3	66.6	11.8	65.1	12.9	0.58
**Gender**									0.99
Female	8368	46.4	1379	43.7	713	52.5	2092	46.4	
Male	9684	53.7	1775	56.3	646	47.5	2421	53.7	
**Comorbidity**									
Diabetes	2496	13.8	567	18.0	275	20.2	842	18.7	< 0.001
Hypertension	8749	48.5	1669	52.9	829	61.0	2498	55.4	< 0.001
Hyperlipidemia	5103	28.3	956	30.3	487	35.8	1443	32.0	< 0.001
COPD	4099	22.7	771	24.5	364	26.8	1135	25.2	< 0.001
CHF	866	4.80	165	5.23	82	6.03	247	5.47	0.06
AF	306	1.70	52	1.65	24	1.77	76	1.68	0.96
CAD	543	3.01	114	3.61	73	5.37	187	4.14	0.001
Treatment									
Chemotherapy	211	1.17	510	16.2	133	9.79	643	14.3	< 0.001

Chi-square test examined categorical data; ^†^*T*-test examined continuous.

**Figure 2:**
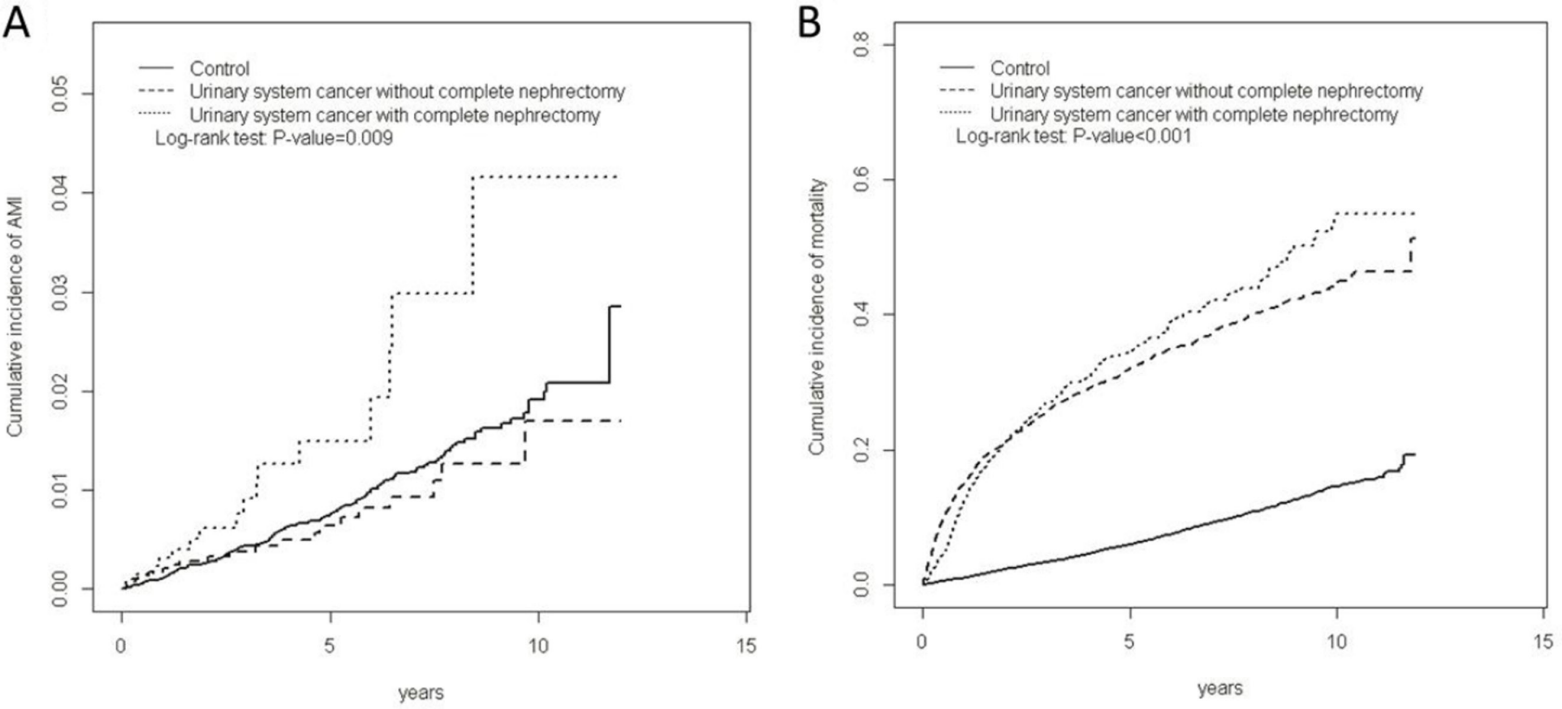
Cummulative incidence of AMI (A) and mortality (B) among three cohorts

The mean follow-up period for AMI in the radical nephroureterectomy, nonnephroureterectomy, and non-UTUC cohorts were 3.47, 4.39, and 5.06 years, respectively. The cumulative incidence of AMI was much higher in patients with UTUC who had undergone radical nephroureterectomy than in those with UTUC who had undergone nephron sparing surgery and those who did not have UTUC (Figure 2A; *P* = 0.009). The overall incidences of AMI were 3.91, 1.44, and 1.70 per 10,000 person-years in the radical nephroureterectomy, nonnephroureterectomy, and non-UTUC cohorts, respectively (Table 2). Multivariable Cox proportional hazard regression analysis for AMI risk revealed that compared with patients in the non-UTUC cohort, those in the radial nephroureterectomy cohort had a significantly higher risk of developing AMI [adjusted HR (aHR) = 1.83, 95% CI = 1.08–3.11]. The age-specific nephroureterectomy to non-UTUC cohorts relative risk of AMI was the higher for the aged ≦ 64 group (aHR = 2.62; 95% CI, 1.03–6.66). The nephroureterectomy patients with comorbidity were associated with significantly higher risk of AMI than the non-UTUC patients (aHR = 2.15; 95% CI, 1.27–3.66).

**Table 2:** Cox models measured incidence densities and hazard ratio of outcome

	Control (*N* = 18052)		Without radical nephroureterectomy (*N* = 3154)		Crude HR *	Adjusted HR^†^	With radical nephroureterectomy (*N* = 1359)		Crude HR	Adjusted HR^†^
	Case	Rate#	Case	Rate#	(95% CI)	(95% CI)	Case	Rate#	(95% CI)	(95% CI)
**AMI**										
All	155	1.70	20	1.44	0.85 (0.53, 1.36)	0.80 (0.50, 1.29)	16	3.39	2.11 (1.26, 3.54)**	1.83 (1.08, 3.11)*
**Age, year**										
≦ 64	31	0.74	5	0.71	0.96 (0.37, 2.46)	0.61 (0.22, 1.68)	6	2.96	4.17 (1.74, 10.0)**	2.62 (1.03, 6.66)*
> 64	124	2.51	15	2.21	0.88 (0.51, 1.50)	0.85 (0.49, 1.46)	10	3.71	1.59 (0.83, 3.04)	1.48 (0.77, 2.84)
**Gender**										
Female	63	1.47	11	1.77	1.20 (0.63, 2.27)	1.06 (0.55, 2.07)	9	3.58	2.55 (1.26, 5.14)**	1.89 (0.91, 3.89)
Male	92	1.89	9	1.18	0.62 (0.32, 1.24)	0.66 (0.33, 1.31)	7	3.17	1.78 (0.83, 3.85)	1.74 (0.80, 3.78)
**Comorbidity**^‡^										
No	27	0.76	3	0.63	0.84 (0.25, 2.76)	1.06 (0.32, 3.50)	0	0.00	-	-
Yes	128	2.30	17	1.87	0.81 (0.49, 1.35)	0.78 (0.47, 1.32)	16	4.69	2.16 (1.28, 3.64)**	2.15 (1.27, 3.66)**
**Stroke**										
All	1293	14.6	188	14.0	0.96 (0.82, 1.11)	1.00 (0.86, 1.17)	69	15.0	1.03 (0.81, 1.31)	0.98 (0.77, 1.25)
**Age, year**										
≦ 64	233	5.64	48	6.91	1.23 (0.90, 1.68)	1.11 (0.81, 1.52)	15	7.42	1.35 (0.80, 2.28)	1.07 (0.63, 1.82)
> 64	1060	22.6	140	21.5	0.96 (0.80, 1.14)	0.94 (0.79, 1.13)	54	20.9	0.93 (0.71, 1.23)	0.93 (0.71, 1.22)
**Gender**										
Female	587	14.2	85	14.2	1.00 (0.80, 1.25)	1.08 (0.86, 1.36)	42	17.3	1.21 (0.88, 1.65)	1.13 (0.82, 1.55)
Male	706	15.0	103	13.8	0.92 (0.75, 1.13)	0.93 (0.75, 1.16)	27	12.4	0.84 (0.57, 1.24)	0.82 (0.56, 1.21)
**Comorbidity**^‡^										
No	209	5.94	29	6.14	1.04 (0.70, 1.53)	1.18 (0.79, 1.76)	8	6.18	1.08 (0.53, 2.19)	1.09 (0.53, 2.22)
Yes	1084	20.4	159	18.2	0.89 (0.76, 1.06)	0.97 (0.82, 1.15)	61	18.4	0.91 (0.70, 1.17)	0.97 (0.75, 1.25)
**Mortality**										
All	1238	13.7	1046	76.5	5.52 (5.09, 6.00)***	4.64 (4.25, 5.06)***	426	91.5	6.37 (5.70, 7.11)***	5.37 (4.80, 6.02)***
**Age, year**										
≦ 64	187	4.52	280	40.1	8.80 (7.31, 10.6)***	5.95 (4.88, 7.26)***	130	64.9	13.6 (10.9, 17.0)***	9.32 (7.36, 11.8)***
>64	1051	21.5	766	114.4	5.25 (4.79, 5.77)***	4.35 (3.94, 4.80)***	296	111.7	4.98 (4.37, 5.67)***	4.54 (3.97, 5.18)***
**Gender**										
Female	452	10.7	466	75.9	7.04 (6.19, 8.01)***	6.18 (5.40, 7.08)***	198	79.7	6.97 (5.90, 8.25)***	5.65 (4.75, 6.72)***
Male	786	16.4	580	77.0	4.66 (4.18, 5.19)***	3.83 (3.41, 4.30)***	228	105.0	6.20 (5.34, 7.19)***	5.23 (4.49, 6.08)***
**Comorbidity**^‡^										
No	282	8.00	277	58.7	7.31 (6.19, 8.62)***	6.35 (5.31, 7.58)***	90	70.2	8.52 (6.72, 10.8)***	6.60 (5.16, 8.44)***
Yes	956	17.4	769	85.9	4.88 (4.44, 5.37)***	4.21 (3.81, 4.66)***	336	99.6	5.46 (4.82, 6.19)***	5.09 (4.48, 5.78)***

PY, person-years; Rate^#^, incidence rate, per 10,000 person-years; Crude HR: relative hazard ratio; Adjusted HR^†^: adjusted hazard ratio controlling for age, sex, and comorbidities of diabetes, hypertension, hyperlipidemia, COPD, CHF, AF, and CAD, and treatment of chemotherapy.

Comorbidity^‡^: Patients with any one of the comorbidities diabetes, hypertension, hyperlipidemia, COPD, CHF, AF, and CAD were classified as the comorbidity group.

*p < 0.05, **p < 0.01, ***p < 0.001.

According to the multivariable analyses, the nonnephroureterectomy cohort did not have a significantly higher risk of AMI (aHR = 0.80, 95% CI = 0.50–1.29) compared with the non-UTUC cohort. The cumulative incidence of mortality was the highest for patients in the radical nephroureterectomy cohort, followed by those in the nonnephroureterectomy and non-UTUC cohorts. Cumulative incidences of mortality differed significantly among the 3 cohorts (Figure 2B, *P* < 0.001).

The incidence mortality rates were 91.5 and 76.5 per 10,000 person-years in the radical nephroureterectomy and nonnephroureterectomy cohorts, respectively, which were significantly higher than that in the non-UTUC cohort (13.7 per 10,000 person-years). Compared with patients in the non-UTUC cohort, those in the radical nephroureterectomy and nonnephroureterectomy cohorts exhibited a 5.37- and 4.64-fold increased mortality risk (95% CI = 4.80–6.02; 95% CI = 4.25–5.06), respectively. The overall incidence and mortality risk in all 3 cohorts were compared after stratification by several variables, such as age, sex, and comorbidity. Mortality risk in all such stratifications was higher in the radical nephroureterectomy and nonnephroureterectomy cohorts than in the non-UTUC cohort.

Table 3 presents a comparison of AMI and mortality risks in the radical nephroureterectomy and nonnephroureterectomy cohorts, which reveals a 2.26-fold higher AMI risk for patients who had undergone radical nephroureterectomy (95% CI = 1.15–4.44). Among patients with comorbidity, the AMI risk was higher in the radical nephroureterectomy cohort than in the nonnephroureterectomy cohort. The mortality risk for patients in the radical nephroureterectomy cohort, relative to that for the patients in nonnephroureterectomy cohort, was the highest in patients belonging to the ≤ 64 years age group (aHR = 1.53, 95% CI = 1.24–1.88). Males exhibited a significantly higher mortality risk after radical nephroureterectomy (aHR = 1.25, 95% CI = 1.07–1.46).

**Table 3:** Comparison of hazard ratio of outcome in urinary system cancer patients with and without radical nephroureterectomy

	Without radical nephroureterectomy	With radical nephroureterectomy	With radical nephroureterectomy
	Reference	Crude HR (95% CI)	Adjusted HR^†^ (95% CI)
**AMI**			
All	1	2.44 (1.26, 4.74)**	2.26 (1.15, 4.44)*
**Age, year**			
≦ 64	1	3.89 (1.18, 12.8)*	3.20 (0.93, 11.04)
> 64	1	1.94 (0.86, 4.39)	1.90 (0.82, 4.40)
**Gender**			
Female	1	2.17 (0.89, 5.30)	1.93 (0.79, 4.71)
Male	1	2.71 (1.00, 7.34)	2.46 (0.87, 6.91)
**Comorbidity**^‡^			
No	1	-	-
Yes	1	2.68 (1.34, 5.36)**	2.69 (1.34, 5.43)**
**Stroke**			
All	1	1.05 (0.79, 1.38)	0.95 (0.72, 1.25)
**Age, year**			
≦ 64	1	1.04 (0.58, 1.86)	0.87 (0.48, 1.57)
> 64	1	0.96 (0.70, 1.31)	0.94 (0.68, 1.30)
**Gender**			
Female	1	1.21 (0.83, 1.76)	1.08 (0.74, 1.57)
Male	1	0.86 (0.56, 1.32)	0.82 (0.53, 1.25)
**Comorbidity**^‡^			
No	1	0.98 (0.45, 2.14)	0.86 (0.39, 1.89)
Yes	1	0.99 (0.74, 1.34)	0.95 (0.70, 1.28)
**Mortality**			
All	1	1.07 (0.96, 1.20)	1.07 (0.96, 1.20)
**Age, year**			
≦ 64	1	1.48 (1.20, 1.82)***	1.53 (1.24, 1.88)***
> 64	1	0.87 (0.76, 0.99)*	0.93 (0.81, 1.07)
**Gender**			
Female	1	0.93 (0.78, 1.09)	0.88 (0.74, 1.04)
Male	1	1.24 (1.06, 1.44)**	1.25 (1.07, 1.46)**
**Comorbidity**^‡^			
No	1	1.12 (0.88, 1.42)	1.03 (0.81, 1.31)
Yes	1	1.03 (0.90, 1.17)	1.08 (0.95, 1.23)

Crude HR: relative hazard ratio; Adjusted HR†: adjusted hazard ratio controlling for age, sex, and comorbidities of diabetes, hypertension, hyperlipidemia, COPD, CHF, AF, and CAD, and treatment of chemotherapy.

Comorbidity^‡^: Patients with any one of the comorbidities diabetes, hypertension, hyperlipidemia, COPD, CHF, AF, and CAD were classified as the comorbidity group.

*p < 0.05, **p < 0.01, ***p < 0.001.

## DISCUSSION

To the best of our knowledge, this is the first population-based cohort study investigating the subsequent risk of AMI and mortality outcomes in patients with UTUC who have undergone radical nephroureterectomy. The results of this study revealed that compared with controls and patients with UTUC who have undergone nephron sparing surgery, AMI risk is greater in patients with UTUC who have undergone radical nephroureterectomy. On stratifying by age, sex, and comorbidities, young age and presence of comorbidities in the radical nephroureterectomy cohort showed higher AMI risk compared with non-nephroureteretomy cohort. The mortality risk was similar in both UTUC cohorts.

Many potential causal links exist between radical nephroureterectomy and cardiovascular outcomes, including adverse traditional and untraditional risk profiles. Prevalence of treated hypertension, diabetes, hyperlipidemia, and congestive heart failure was higher in the radical nephroureterectomy cohort than in the nonnephroureterectomy and non-UTUC cohorts. However, after adjustment for these comorbidities, the AMI risks remained higher in the radical nephroureterectomy cohort than in the nonnephroureterectomy cohort, which suggests the existence of untraditional cardiovascular risk factors and related casual pathways.

CKD status after radical nephroureterectomy is a possible explanation for the higher AMI risks in the study cohorts. Anavekar et al. reported that patients with CKD had higher risk of subsequent cardiovascular events than do individuals with normal renal function [16]. Several hypotheses have been postulated on the relationship between CKD status after radical nephroureterectomy and cardiovascular events. Patients with CKD have higher levels of homocysteine, inflammation, and oxidative stress, possibly causing endothelial dysfunction; smooth muscle proliferation; platelet aggregation; and activation of coagulation factors V, X, and XII, providing a prothrombotic environment [7, 17]. In addition, patients with CKD tend to have higher aortofemoral pulse wave velocity and carotid intima thickness, which substantially affect the development of cardiovascular events [18, 19]. To eliminate the bias of CKD which often existing in previous studies, we completely exclude those who had CKD diagnosis before and after radical nephroureterectomy or nephron sparing surgery. Our results demonstrated a clear relationship between radical nephrouretectomy and AMI.

During our follow-up period, no statistical difference was observed for mortality risk between the UTUC cohorts, but the risk in both the cohorts was higher than that in the non-UCUT cohort. This finding suggests that in patients with UTUC, the cancer itself and its associated sequelae more strongly influences mortality outcomes than cardiovascular events do. Our hypothesis is also strengthened by our stratification analysis, which revealed that the mortality rate in patients with UTUC and cardiovascular comorbidities and that in patients with UTUC alone did not differ significantly.

This study has several strengths. First, the NHIRD is a population-based, long-term, and comprehensive database of the medical records of each Taiwan resident enrolled in the NHI program. Adapting the NHIRD enabled the longitudinal follow-up of patients with UTUC who had undergone radical nephroureterectomy or nephron sparing surgery and the subsequent evaluation of their risks of cardiovascular events. Second, the study cohorts were frequency matched by age, sex, cardiovascular risk factors and previous CAD to increase the validity of this study. However, a few limitations affect the present study. First, the NHIRD does not contain detailed information on the severity, stage, grade, and location of UTUC; smoking and dietary habits; and family history, all of which are potential confounding factors in the development of cardiovascular events. Furthermore, such clinical variables as the levels of creatinine, low-density cholesterol, high-density cholesterol, blood pressure, blood glucose, and hormone usage are unavailable in the database. We had also no information about operation time, operative position, and status of blood transfusion in NHIRD which would also affect the risk of AMI. There was also lack of the tumor-staging for UTUC which was indicated for radical nephroureterectomy or not in NHIRD. Thus, possible indication bias would exist. Nevertheless, we used ICD-9-CM codes to identify the following comorbidities: hypertension, diabetes, hyperlipidemia, congestive heart failure, and atrial fibrillation. The accuracy of diagnosis is strictly verified by the NHI to prevent errors and overutilization of medical resources.

In conclusion, patients with UTUC who have undergone radical nephroureterectomy are at a higher risk of developing AMI compared with those receiving nephron sparing surgery, but the mortality risk is similar. Considering the higher AMI risk in patients who have undergone radical nephroureterectomy, close monitoring, lifestyle modification, and medical intervention is essential for these high-risk patients.

## MATERIALS AND METHODS

### Data sources

The National Health Insurance Research Database (NHIRD) contains original claims data from the NHI program. The Taiwan NHI program, launched in 1995, covers > 99% of the 23.74 million residents of Taiwan [20]. Details of this NHIRD have been previously documented [21, 22]. To link the data of the insured individuals, each individual is assigned a deidentified identification number by the National Health Research Institutes. We used this number to link 3 subset databases of the NHIRD, namely the Registry of Catastrophic Illnesses Patient Database (RCIPD), Longitudinal Health Insurance Database 2000, and Registry of Beneficiaries. International Classification of Diseases-9-Clinical Modification (ICD-9-CM) codes were used to define diseases in the NHIRD.

### Ethics statement

The NHIRD encrypts patient personal information to protect privacy and provides researchers with anonymous identification numbers associated with relevant claims information, including sex, date of birth, medical services received, and prescriptions. Therefore, patient consent is not required to access the NHIRD. This study was approved to fulfill the condition for exemption by the Institutional Review Board (IRB) of China Medical University (CMUH104-REC2-115-CR1). The IRB also specifically waived the consent requirement.

### Study subjects

Data from the RCIPD was used to identify patients who were diagnosed with UTUC (ICD-9-CM code 189) between 2000 and 2010. Patients aged ≥ 20 years with UTUC who had undergone nephroureterectomy (ICD-9-OP 55.5) were classified into the radial nephroureterectomy cohort. Patients with UTUC who had not undergone nephroureterectomy were grouped into the nonnephroureterectomy cohort. The index date in the radial nephroureterectomy cohort was set as the date on which nephroureterectomy was performed. The same index date was set for patients in the nonnephroureterectomy (i.e., nephron sparing surgery) cohort. Patients with a history of acute myocardial infarction (AMI) (ICD-9-CM code 410), stroke (ICD-9-CM codes 430–438) prior to the index date, history of chronic kidney disease (ICD-9-CM code 585), or missing age or sex information were excluded from the study. For each patient with UTUC, 4 non-UTUC individuals were randomly selected from the pool of individuals without AMI and stroke at the baseline. They were frequency matched by the year of index date, age (5-year spans), and sex. These patients were grouped into the non-UTUC cohort. The study patients were followed up until the diagnosis date of outcomes such as AMI, stroke diagnosis, or mortality; withdrawal from the NHI program; or December 31, 2011.

### Diagnostic criteria of comorbidities and outcomes

Preexisting comorbidities included diabetes (ICD-9-CM code 250), hypertension (ICD-9-CM codes 401–405), hyperlipidemia (ICD-9-CM code 272), chronic obstructive pulmonary disease (COPD) (ICD-9-CM codes 491, 492, and 496), congestive heart failure (CHF) (ICD-9-CM codes 428), atrial fibrillation (AF) (ICD-9-CM codes 427.31 and 427.32), and CAD (ICD-9-CM codes 414.01), atherosclerosis (ICD-9-CM codes 440.30), PAD ((ICD-9-CM codes 443.9), or prior MI (ICD-9-CM codes 412.0). In this study, we enrolled patients only who had coding of comorbidities in the first three principle diagnosis and more than three times. The diagnoses of these comorbidities were based on the corresponding ICD-9 codes which were judged and determined by related specialists and physicians according to the standard clinical criteria. All insurance claims should be scrutinized by medical reimbursement specialists and peer review according to the standard diagnosed criteria in the study. If these doctors or hospitals make wrong diagnoses or coding, they will be punished with a lot of penalties. Therefore, the diagnoses used in this study should be correct and reliable. The accuracy of diagnostic codes of AMI (ICD-9-CM code 410) in NHIRD has been validated [23].

### Statistical analysis

The distribution of demographic factors and comorbidities in the UTUC (radial nephroureterectomy and nonnephroureterectomy cohorts) and non-UTUC cohorts were compared using chi-square test and Student’s *t*-test. The Kaplan–Meier method was employed to determine the cumulative incidence curves of AMI and mortality in the 3 cohorts, and log-rank test was used to examine the difference among the curves. The overall rate and age, sex- and comorbidity-specific incidence rates of AMI and mortality (per 10,000 person-years) were calculated for each cohort. The overall risk and age-, sex-, and comorbidity-specific risk of developing AMI and mortality associated with radical nephroureterectomy were calculated using univariable and multivariable Cox proportional hazard regressions models. Hazard ratios (HRs) and 95% confidence intervals (CIs) were estimated in the Cox model. All statistical analyses were performed using SAS software Version 9.4 (SAS Institute, Inc., Cary, NC, USA), and *P* < 0.05 was considered statistically significant.
